# Role of PD-1/PD-L1-mediated tumour immune escape mechanism and microsatellite instability in the BCG failure of high-grade urothelial carcinomas

**DOI:** 10.55730/1300-0144.5526

**Published:** 2022-10-09

**Authors:** Fadime Gül SALMAN, Duygu KANKAYA, Hilal ÖZAKINCI, Yasemin ŞAHİN, Eralp KUBİLAY, Evren SÜER, Serhat HAYME, Sümer BALTACI

**Affiliations:** 1 Department of Pathology, Faculty of Medicine, Ankara University, Ankara, Turkey; 2 Department of Urology, Faculty of Medicine, Ankara University, Ankara, Turkey; 3 Department of Biostatistics, Faculty of Medicine, Ankara University, Ankara, Turkey

**Keywords:** Nonmuscle-invasive bladder carcinoma, microsatellite instability, mismatch repair, PD-L1, immunotherapy, BCG therapy

## Abstract

**Background/aim:**

Intravesical BCG treatment fails inexplicably in 30%–45% of patients for high-grade nonmuscle-invasive bladder cancer (NMIBC). We aimed to investigate the role of PD-1/PD-L1 interaction on BCG failure of high-grade NMIBC and to identify biomarkers for predicting BCG responsive cases.

**Materials and methods:**

Thirty BCG responsive and 29 nonresponsive NMIBCs were included in the study. Expressions of PD-L1(SP-263), MSH2, MSH6, PMS2, and MLH1 were evaluated on pre- and post-BCG transurethral resection (TUR-B) specimens by immunohistochemistry. PD-L1(SP-263) expression was categorised as negative/low, high. DNA mismatch repair protein (MMR) expressions were classified as “reduced” if ≤30% of nuclei stained, “preserved” if >30% of nuclei stained. Microsatellite instability (MSI) testing was performed by PCR using five mononucleotide markers.

**Results:**

Reduced DNA MMR protein expression was found to be significantly higher in the pretreatment biopsies of BCG-responsive group than the BCG nonresponsive tumour group (p = 0.022). PD-L1 expression did not show any significant difference between the pre- and posttreatment TUR-B specimens of the BCG nonresponsive tumour group or between the pretreatment TUR-B specimens of BCG nonresponsive and the BCG responsive groups (p = 0.508, p = 0.708, respectively).

**Conclusion:**

Immune escape of tumour cells by PD-1/PD-L1 interaction does not seem to have any role in BCG failure of NMIBCs. Reduced MMR expression may help to determine cases that will respond well to BCG therapy. A better antitumour activity of BCG in NMIBCs with reduced MMR expression may be related to the ongoing accumulation of cancer neoantigens in correlation with increased tumour mutation load as a result of DNA repair defects.

## 1. Introduction

Bladder cancer is the 9th most common malignancy worldwide [1]. Most of them (70%–80%) are nonmuscle-invasive urothelial carcinomas and conservative approaches are applied to prevent tumour recurrence or progression [2]. Instillation of intravesical Bacillus Calmette-Guerin (BCG) following transurethral resection is the gold standard treatment approach for high-risk nonmuscle-invasive bladder carcinoma (NMIBC) [3,4]. However, this treatment fails in 30%–45% of patients, and the lack of another effective conservative second-line treatment option for this patient group raises the requirement of radical cystectomy, which is a radical therapeutic option with significant morbidity and mortality rates [3]. This leads to search biomarkers to predict BCG response or alternative conservative therapies for BCG nonresponders.

After intravesical instillation of BCG, it binds to the urothelial cells via extracellular matrix glycoproteins such as fibronectin and integrins. It is followed by cellular internalization. This leads to the induction of innate immunity by cytokines such as interleukin-1 (IL-1), interleukin-2 (IL-2), interleukin-6 (IL-6), interleukin-8 (IL-8), tumour necrosis factor- α (TNF-α), interferon-ɣ (IFN-ɣ) [4]. In addition to this nonspecific inflammatory response, a more specific, adaptive immune response is triggered by the antigen presentation to T cells [5]. The antitumour effects of BCG depend on the activation of both CD4+ and CD8+ T cells [4,6].

Programmed death-1 (PD-1) is expressed on activated T, B, and myeloid cells. PD-L1 (B7-H1) and PD-L2 (B7-DC) are two ligands of PD-1. PD-L1 is broadly expressed on T and B cells, other immune cells, epithelial, endothelial, and tumour cells. Engagement of PD-1 by PD-L1 leads to the inhibition of T-cell-mediated immune responses via inhibition of the Ras-Raf-MEK-ERK and PI3K-AKT cascades in T cells [7]. In malignancies, tumour cells express PD-L1 to escape from the immune response [7]. Increased expression of PD-L1 on tumour cells or tumour infiltrating lymphocytes (TILs) leads to exhaustion of T cells. Immune checkpoint inhibitors (ICIs) that block PD-1/PD-L1 interaction reactivate the T cell response against tumour cells [8].

Failure in BCG treatment evokes a hypothesis that tumour cells may develop some mechanisms to escape from the immune system and this raises the question of whether PD-1/PD-L1 interaction has a role in this immune escape mechanism and whether the blockage of this pathway may be a solution for BCG nonresponders.

The DNA “mismatch” repair (MMR) system repairs errors that occur during DNA replication and sustains mutation rates at a low level. In eukaryotes, the DNA MMR system has two heterodimers consisting of MutS protein homologue 2 (MSH2)/MutS protein homologue 6 (MSH6) and MutL homologue 1 (MLH1)/PMS1 homologue 2 (PMS2) [9]. DNA MMR proteins recognize the mismatched nucleotide in the newly synthesized strand and then remove the mismatched nucleotide [10]. There are regions called “microsatellites” where DNA polymerases are more prone to make mistakes. Microsatellites are defined as repeats (usually 10–60 times) of the same base or sequence of bases, ranging in length from one to six or more base pairs. Thus, defects in the DNA MMR system cause DNA strands with mismatched nucleotides, the status of high microsatellite instability (MSI-H), and the increased mutational burden in such tumours create neoantigens which cause more immune response. Therefore, MSI-H tumours have significant upregulation of PD-1 and PD-L1, enabling tumour cells’ immune escape and ICI therapy (anti-PD1, anti-PD-L1) is found more effective in MSI-H cases [9].

This study aimed to investigate the role of PD-1/PD-L1 interaction on BCG failure of high-grade NMIBC and to identify biomarkers for predicting NMIBC cases that will not respond to BCG therapy by focusing on the MSI status/DNA MMR gene expression of these tumours. This is the first study in the literature that deals with all these parameters together in BCG nonresponsive cases.

## 2. Material and methods

### 2.1. Patients and samples

**Figure 1:**
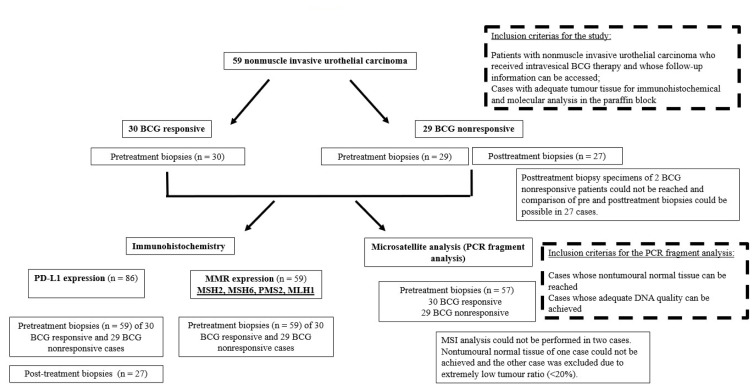
Flow chart illustrating the inclusion and exclusion criteria of cases in the study.

Our study was designed retrospectively and included 59 nonmuscle invasive high-grade urothelial carcinoma cases (Ta, T1), who were diagnosed and followed up at Ankara University, School of Medicine, Departments of Pathology and Urology, between 2005 and 2018. All the patients had received intravesical BCG therapy and the median follow-up period of the patients was 53 (range 12 to 156) months. “BCG nonresponsive” patient group was classified according to the criteria specified in the European Association of Urology Guidelines in 2020 [11] as follows: 21 BCG-refractory tumours, 7 progressive tumours (muscle-invasive bladder cancer at any time after TUR-B), 1 BCG-relapsing tumour (recurrence of high-grade tumour after BCG maintenance). In the BCG nonresponsive group, both pretreatment and posttreatment TUR-B specimens; in the BCG responsive group, only pretreatment TUR-B specimens were taken into consideration. However, posttreatment TUR-B specimens of 2 BCG nonresponsive patients could not be reached. Inclusion and exclusion criteria of the cases were detailed in the flow chart (Figure 1).

Hematoxylin and eosin (H&E)-stained sections were reviewed according to the tumour type, histological variant, histological grade (low, high), degree of nuclear pleomorphism (mild, prominent), the extent of invasion (1 focus: focal, >1 focus: multifocal), long diameter of largest invasion focus (<2mm, ≥2mm), depth of lamina propria invasion determined according to the muscularis mucosa invasion (superficial lamina propria, deep lamina propria invasion), presence of carcinoma in situ, and density of tumour-associated inflammatory cells (IC). Tumours were graded according to WHO 2016 grading system. And then, formalin-fixed paraffin-embedded (FFPE) blocks that best represent the tumour were selected for immunohistochemistry (IHC) and polymerase chain reaction (PCR) based MSI testing.

### 2.2. Immunohistochemistry

H&E staining and immunostaining were performed on 4 μm thick consecutive sections. All immunohistochemical staining was carried out on a Benchmark Ultra System (Ventana Medical Systems, Tucson, AZ, USA) with antibody visualization using the OptiView DAB IHC Detection Kit (Ventana Medical Systems) for PD-L1, PMS2, and UltraView DAB IHC Detection Kit (Ventana Medical Systems) for MSH2, MSH6, MLH-1 according to the manufacturer’s instructions. All IHC stained slides and corresponding H&E-stained slides were digitally scanned [3DHistech Panoramic 250 Flash (3DHISTECH Ltd., Budapest, Hungary)].

#### 2.2.1. Evaluation of PD-L1 expression

Expression of PD-L1 was evaluated by IHC using rabbit monoclonal anti-PD-L1 clone SP263 [Ventana Medical Systems, Inc., Tucson, Arizona, USA; retrieval: EDTA 60’; incubation: 120’; ready to use [RTU] dilution]. Human placental tissue was utilized as a positive control. As we described below, PD-L1 stained sections were evaluated by using assay scoring algorithms that were published for PD-L1 clone SP263 [12].

Initially, H&E-stained sections were screened to determine the immune cell proportion (ICP) for each slide which was the percentage of the tumour area occupied by any tumour-associated immune cells. Immune cells adjacent to the tumour and infiltrating the tumour (IC) were taken into consideration. And cases were classified into three groups concerning ICP (group 1: ≤ 5%, group 2: 6%–10%, group 3: (>10%).

**Figure 2:**
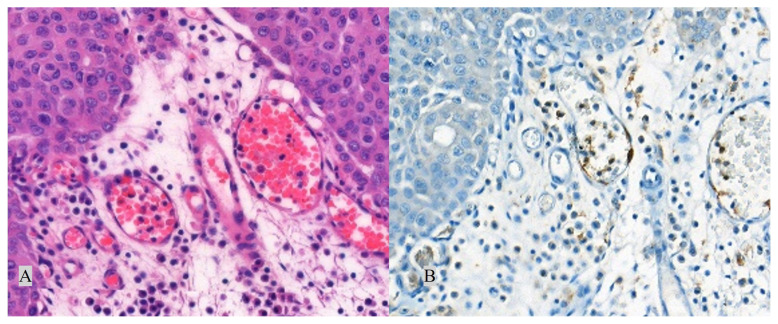
Negative/low (<25%) PD-L1 expression. Corresponding H&E (A, 400×) and PD-L1 (B, 400×) stained sections. Arrow: Positive staining in immune cells.

**Figure 3:**
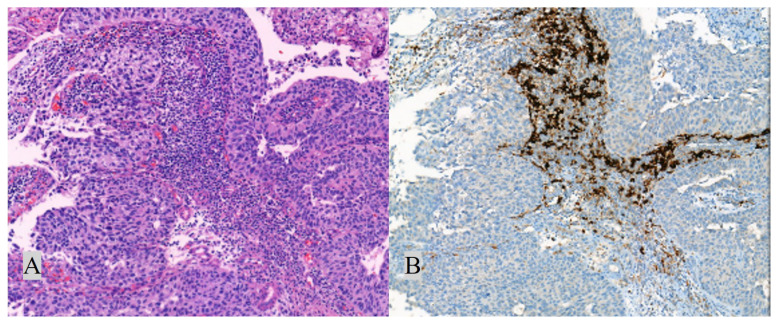
High (≥25%) PD-L1 expression. Corresponding H&E (A, 200×) and PD-L1 (B, 200×) stained sections. Arrow: Positive staining in immune cells.

PD-L1 status was determined by the percentage of tumour cells (TC+) with any membranous staining (partial or complete) or by the percentage of tumour-associated immune cells with membranous/cytoplasmic/punctate staining (IC+). IC+ and TC+ were scored as deciles and quartiles and categorised as “negative/low” (Figures 2A and 2B) and “high” (Figures 3A and 3B) expressions. PD-L1 expression was classified as “high”, if any of the followings were met: ≥25% of TC exhibit membranous staining; or ICP > 1% and IC+ ≥ 25%; or ICP = 1% and IC+ = 100%. Conditions other than these were classified as negative/low PD-L1 expression.

#### 2.2.2. Evaluation of DNA MMR proteins expression

**Figure 4:**
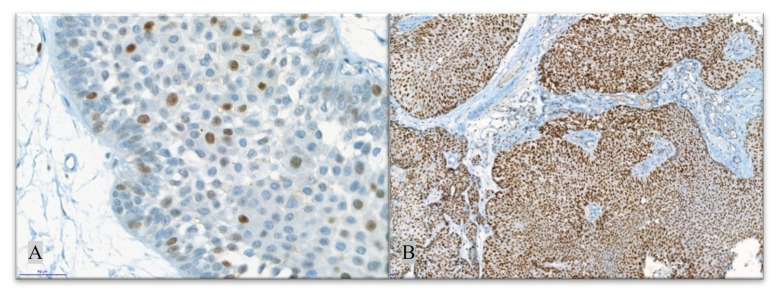
Reduced (≤30%) expression of DNA MMR protein (MSH6, 400×) (A) and preserved (>30%) expression of DNA MMR protein (MSH2, 100×) (B). Arrow: Positive staining in tumour cells.

DNA MMR family include several proteins. We evaluated the expressions of 4 major MMR proteins -MLH1, MSH2, PMS2, MSH6- by immunohistochemistry, since these proteins are the most commonly studied of DNA MMR family and have been found to be highly associated with microsatellite instability. Expression of DNA MMR proteins was investigated by IHC using MLH1 (mouse monoclonal primary antibody; clone M1; retrieval: EDTA 60’; incubation: 48’; ready to use [RTU] dilution), MSH2 (mouse monoclonal primary antibody; clone G219–1129; retrieval: EDTA 60’; incubation: 40’; ready to use [RTU] dilution), MSH6 (mouse monoclonal primary antibody; clone 44; retrieval: EDTA 90’; incubation: 56’; ready to use [RTU] dilution), and PMS2 (rabbit monoclonal primary antibody; clone EPR3947; retrieval: EDTA 60’; incubation: 60’; ready to use [RTU] dilution). Normal colonic mucosa was utilized as a positive control. Each digitally scanned slide was reviewed and then the percentage of positively stained TC was determined. Inevaluable areas due to necrosis or cautery/crush artifacts were disregarded. Lymphocytes and some stromal cells served as the internal positive control. The immunostaining was repeated for cases without internal control staining (13 cases). Immunostainings for each DNA MMR proteins were classified as “absent” if the case had no positively (0%) stained TC nuclei, “reduced” (Figure 4A) if ≤30% of TC nuclei stained positively, and “preserved” (Figure 4B) if >30% of tumour cell nuclei stained positively [13,14].

### 2.3. MSI testing

#### 2.3.1. DNA extraction

DNA was extracted from 5 μm thick FFPE tissue sections. To enrich tumour DNA, macrodissection was applied to slides with areas containing less than 50% tumour cells. MSI testing was performed on 57 patients. In 2 cases (both BCG refractory), there was insufficient material (<20% tumour cells) to extract enough DNA to perform the analysis. For DNA extraction from FFPE tissues, the QIAamp tissue kit (Qiagen Inc, Santa Clarita, CA) was used according to the manufacturer’s instructions. The corresponding normal control DNA was derived from the nontumoural tissue of each patient.

#### 2.3.2. PCR-based MSI testing

Normal and tumour DNA pairs were analysed for each case by PCR-based commercial MSI testing kit developed by Promega (MD1641 MSI Analysis System, Version 1.2). This kit consists of 5 poly-A mononucleotide markers (BAT-25, BAT-26, NR-21, NR-24, and MONO-27) and two highly polymorphic pentanucleotide repeat markers (Penta C and Penta D). The mononucleotide markers are used for MSI determination, and the pentanucleotide markers are used to detect potential sample mix-ups and/or contamination. PCR products were analysed by capillary electrophoresis using ABI 3500 Genetic Analyzer (Applied Biosystems, Forster City, CA). Microsatellite region lengths were compared between paired normal and tumour tissue for each patient.

#### 2.3.3. Microsatellite analysis system with Promega panel

**Figure 5:**
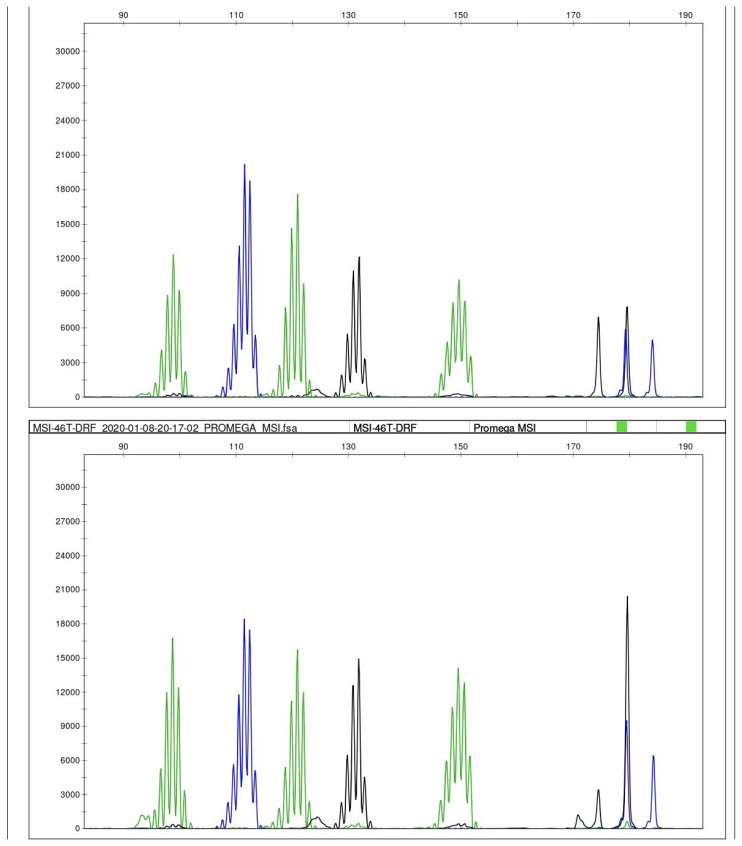
MSI testing. Length distribution of microsatellite regions on five mononucleotide repeat loci (NR-21, BAT-26, BAT-25, NR-24, MONO-27) were compared between tumour (as shown below) and matched normal tissue (as shown above). No instability was detected in any locus in any of the cases. All cases were microsatellite stable (MSS).

We interpreted MSI testing as follows: microsatellite instability at ≥2 mononucleotide loci as MSI-high (MSI-H), instability at a single mononucleotide locus as MSI-low (MSI-L), and no instability at any of the loci as microsatellite stable (MSS) (Figure 5) [15].

### 2.4. Statistical analysis

All analyses were performed by using SPSS software v.20.0 (IBM, Armonk, NY, USA). Shapiro-Wilks test was used to assess the assumption of normality. Continuous variables that do not have normal distribution were expressed as median (minimum-maximum). Also, categorical variables were summarised as counts (percentages). For nonnormally distributed continuous variables, differences between groups were tested using Mann-Whitney U test. For pre- and posttreatment comparisons in BCG nonresponsive group, the Wilcoxon sign rank test was used for continuous variables, while McNemar’s test was performed for categorical variables. Associations between categorical variables were determined by chi-square analysis or Fisher’s exact test, while associations between continuous variables were determined by Pearson correlation analysis. p-values of less than 0.05 were considered statistically significant.

## 3. Results

### 3.1. Patient characteristics

The mean age of the patients was 67.74 (48–85) years [69.73 (52–85) years in BCG responsive group and 66.34 (48–84) years in BCG nonresponsive group; p = 0.139]. The clinicopathological features are summarised in Table 1.

Multifocality was more in the BCG nonresponsive group (Table 1; p=0.001). 22 (75.9%) of 29 BCG nonresponsive patients had multiple tumour foci. BCG failure was higher in the group with deep lamina propria invasion (shown in Table 1; p=0.038).

ICP showed a difference neither between pretreatment biopsies of the BCG nonresponders and the BCG responders nor the pre- and posttreatment biopsies of the BCG nonresponders (p=0.922 and p=0.352, respectively). There was not any statistically significant difference between BCG responders and BCG nonresponders among pT1 patients, in terms of the extent of tumour invasion (focal: 1 focus, multifocal: >1 focus) or long diameter of largest invasive focus (<2mm, ≥2mm). ICP was lower in tumours with concomitant carcinoma in situ (CIS) (n:10) compared to cases without CIS (n:49) (p=0.018).

### 3.2. PD-L1 expression

All biopsies showed negative/low PD-L1 expression in TC. The distribution of ICP groups and PD-L1 expression of IC in pretreatment biopsies of both BCG nonresponsive and BCG responsive cases are summarised in Table 2.

PD-L1 expression did not show any significant difference between the pre- and posttreatment TUR-B specimens of the BCG nonresponder group (shown in Table 3) or between the pretreatment TUR-B specimens of BCG nonresponder and the BCG responder groups (p = 0.508, p = 0.708, respectively).

**Figure 6:**
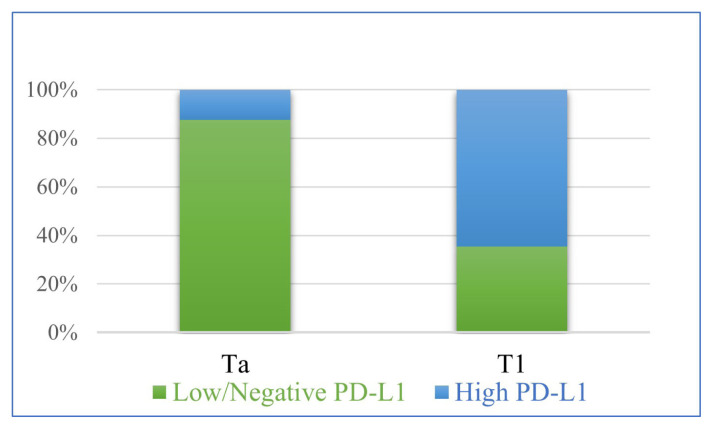
Comparative scheme of PD-L1 expression in IC in stage Ta and T1 urothelial carcinoma cases (n: 59; p = 0.008).

High PD-L1 expression in IC was found more common in T1 tumours than Ta tumours (Figure 6; p = 0.008). A positive correlation was observed between the percentage of ICP and PD-L1 expression in IC (Pearson correlation: 0.428; p = 0.001). There was no relation between PD-L1 expression in IC and the histopathologic features evaluated (depth of lamina propria invasion, long diameter of largest invasive focus, and the extent of lamina propria invasion or the presence of concomitant carcinoma in situ) (p = 0.313; p = 0.859; p = 0.603, p = 1.000, respectively).

### 3.3. DNA MMR protein expressions

Reduced DNA MMR protein expression was found to be significantly higher in the pretreatment biopsies of BCG-responsive group than the BCG nonresponsive tumour group (p = 0.022). Reduced expression of DNA MMR proteins was detected in 24 (40.7%) pretreatment TUR-B specimens and 17 (70.8%) of 24 cases were BCG responders (detailed in Table 4; p = 0.022). We did not observe the total loss of DNA MMR proteins in any case.

Cases with reduced expression of DNA MMR proteins were as follows: isolated MSH6 (n = 11), isolated PMS2 (n = 6), both MSH6 and PMS2 (n = 5), combined MSH2 and MSH6 (n = 1), and one case with reduced expression of MSH2, MSH6, and PMS2. No subclonal staining loss (tumour area with abrupt loss of staining) was observed in any of the cases with reduced DNA MMR protein expression.

There was no relation between reduced expression of DNA MMR proteins and increased rate of ICP or high PD-L1 expression (p = 0.778, p = 0.891, respectively).

### 3.4. MSI testing

Two of the cases were not included in the microsatellite analysis. One of the cases did not have any normal tissue and the other one had an extremely low tumour ratio (<20%). MSI testing was performed in samples of 57 patients and all cases were microsatellite stable (MSS).

## 4. Discussion

Recently, it has been shown that tumour cells can escape from the antitumour response of the immune system by using the mechanisms that protect normal tissues from the immune system. Immune checkpoint inhibitor (ICI) treatments, in which these mechanisms are blocked, have come up as alternative treatment approaches in various cancer types. Antibodies that block proteins in this pathway are intended to increase the effectiveness of T cell response against the tumour cells. Blockage of these immune escape mechanisms by monoclonal antibodies has shown to be a promising method in the treatment of many malignancies, including advanced urothelial cancer [16]. Since the antitumour effect of BCG occurs with the activation of T cells against tumour cells, an immune escape mechanism similar to that described above may also underlie BCG unresponsiveness. If this mechanism is found to be implicated in the BCG unresponsiveness, antibodies that block proteins in this pathway can be used in combination with BCG therapy to increase the effectiveness of it, or ICIs can promise hope for BCG unresponsive urothelial carcinomas as an alternative treatment approach.

On the other hand, MSI is known to be a biomarker in determining the response to the immune checkpoint inhibitors [9]. It has been reported that MSI-H tumours have increased mutation load due to the MMR defects and this results in a more intense immune response. Thus MSI-H tumours show higher PD-L1 expression and they have shown to be suitable candidates for ICI therapy [9,17,18].

In the present study, we investigated whether PD-L1 mediated immune escape mechanisms have any role on the BCG unresponsiveness of NMIBCs and whether status of MSI/MMR defect predicts BCG response of this tumour group.

In bladder carcinoma, the rate of PD-L1 positivity in tumour cells ranges from 4.3% to 100% in published studies [19–24]. Usage of the different PD-L1 clones or thresholds of positivity and the varying cell type in which immunostaining was evaluated could be the cause of different results in previous studies. This leads to difficulties in the interpretation and comparison of the results of these studies. In this study, PD-L1 clone SP263, which is in use to evaluate the clinical applicability of durvalumab, one of the Food and Drug Administration (FDA)-approved ICIs for the treatment of bladder carcinomas, was used. In our study, none of the cases showed high PD-L1 expression in TCs. We found a positive correlation between the percentage of ICs and PD-L1 expression of ICs and high PD-L1 expression in ICs was more frequent in T1 tumours than Ta tumours (p = 0.008) in accordance with the results in the literature [16,20,22,24–26]. This finding suggests that the PD-1/PD-L1 mediated immune escape mechanism of tumour cells may have a role in the progression of bladder carcinomas.

Comparison between pretreatment and posttreatment biopsies of BCG nonresponsive patients showed no difference in terms of IC ratio and this finding showed that the targeted immune response by BCG instillation could not be achieved in these patients.

Based on the question of whether the BCG unresponsiveness in NMIBCs is a result of the immune escape of the tumour cells by PD-1/PD-L1 interaction, we made an investigation by two ways. We compared PD-L1 expression between pretreatment biopsies of BCG-responsive and BCG-unresponsive groups of NMIBCs and between pretreatment and posttreatment biopsies of BCG unresponsive NMIBCs. There are different results in the literature regarding this issue. In one study, increased PD-L1 expression was detected in posttreatment biopsies [21]. However, there are also studies reporting that PD-L1 expression decreased after the instillation of BCG [22,26] or that there was no relationship between PD-L1 expression and BCG treatment [16,19,25]. None of them, but two, made a comparative assessment of PD-L1 expression between pretreatment and posttreatment biopsies, specifically within the tumour group with BCG failure. One of them reported widespread and strong PD-L1 expression in BCG granuloma areas in recurrence biopsies of BCG refractory cases [23]. In the other more recent study, posttreatment PD-L1 expression (with Clone: SP142, Ventana) levels on IC significantly decreased after BCG immunotherapy in patients with refractory recurrence, while it remained stable in patients who upstaged to muscle-invasive disease or subsequently developed metastasis after BCG treatment. Hence, as a result of this study, it has been reported that the salvage anti-PD-L1 therapy following BCG failure might be limited in this patient group with BCG refractory recurrence. [26]. In our study, there was no difference between pretreatment and posttreatment PD-L1 expression of ICs or TCs in cases with BCG failure. The absence of the expected increase in PD-L1 expression after treatment indicated that the PD-1/PD-L1 mediated immune escape mechanism does not have any role in BCG failure. In a very recently published study of BCG-responder and nonresponder urothelial carcinoma in situ cases, no difference in terms of PD-L1 SP263 or SP142 clone expression between the BCG responder and BCG-unresponsive patient group was found, but PD-L1 22C3 clone expression of tumour cells was found to be increased in posttreatment biopsies of BCG unresponsive cases and it has been suggested that PD-L1 22C3 expression may identify CIS cases that fail the BCG therapy [27].

Historically, MSI was first identified in tumours of patients with Lynch syndrome. Urothelial carcinoma in the upper urinary tract occurs 14 times more frequently in Lynch syndrome families than the normal population, but there is no such relationship for urothelial carcinoma of the bladder [28]. In studies investigating the frequency of MSI in urothelial carcinoma of the bladder, various (mono-, di- tri- or tetranucleotide) panels have been used, and the frequency of MSI has been reported between 0% and 100% [13,29–37]. These variations are likely due to the differences in microsatellite panels, criteria of MSI-H, and properties of the patient population [37]

Immunohistochemistry is well correlated with the MSI phenotype and can be used to determine MSI status, as a quick and cheap method. In colourectal carcinomas, tumours that have nuclear staining with MMR proteins, even focal/heterogeneous, are evaluated as MSS [38].

But it has recently been shown that microsatellite instability is not always a homogeneous event throughout the tumour in sporadic colourectal carcinomas. Joost et al. categorised the heterogeneous MMR protein expression of colourectal carcinomas in three subtypes: intraglandular (staining loss within or in-between glandular structures), clonal (staining loss in whole glands or groups of glands), and compartmental (staining loss in larger tumour areas/compartments or between different tumour blocks). They performed MSI analysis separately both in tumour areas with intact MMR expression and with loss of expression and, MSI-H was detected only in tumour areas with loss of MMR expression. [39]. This finding revealed the subclonal MSI-H status in tumours, and brought up the need for MSI analysis including areas only with the loss of MMR expression for more accurate results in MSI analysis of tumours [39,40].

There are conflicting results of studies investigating the relationship between MMR protein expression and MSI status in bladder carcinomas. A previous study reported that the reduced expression of MMR proteins in urothelial carcinomas of the bladder correlated with MSI [29], conversely, some publications reported that there was no correlation between reduced expression and MSI [13,33]. In the present study, none of the cases had a total loss of expression of MMR proteins by IHC, but reduced expression of DNA MMR proteins was observed in 40.7% of total cases. Since reduced / heterogeneous MMR protein expression in our cases was of intraglandular heterogeneity type, MMR positive and negative cells were intertwined, and it could not be possible to perform an isolated PCR analysis by focusing only on MMR negative cells. Further analysis by a technique of single-cell capturing in these areas would enable us to determine the MSI status of these MMR negative cells; however, we could not have the chance of this advanced technique.

MSI is known to be a biomarker in determining the response to the ICIs [9]. It has been shown that there is a correlation between MSI-H status and PD-L1 expression in MSI-H tumours, such as gastrointestinal and endometrial carcinomas [17,18]. However, some studies reported that there is no such correlation in tumours where MSI is rarer, such as breast and lung cancer [41,42]. In compatible with that, there was not any correlation between reduced MMR expression and the rate of ICP or PD-L1 expression in the present study, as all cases with reduced expression of MMR protein were MSS by PCR fragment analysis.

Recently, tumour mutation burden, neoantigen load, and the presence of mutations in DNA damage response genes have been identified as promising biomarkers of benefit from immunotherapy with immune checkpoint inhibitors in several malignancies, including urothelial carcinoma [43]. The presence of deleterious mutations in DNA damage response genes was also reported to be associated with improved outcomes after BCG immunotherapy and tumour mutation burden, high neoantigen load were found to be higher in the BCG-responsive group compared to BCG-unresponsive patients. In the present study, we investigated the role of DNA MMR gene expression, a gene group that is also involved in the DNA damage response, in predicting the response of NMIBC cases to BCG therapy and the expression of DNA MMR genes were evaluated at the protein level by using immunohistochemistry, which is an easier and cheaper method. As a prominent finding of this study, a statistically significant correlation between reduced DNA MMR protein expression and the responsiveness to BCG therapy was detected (p = 0.022). Our study is the first to investigate the relationship between DNA MMR gene expression status and response to BCG therapy in NMIBC cases receiving BCG therapy and it can be suggested that the evaluation of DNA MMR protein expression may be useful in predicting NMIBC cases that will respond well to BCG therapy. Since there is no validated predictive biomarker to guide patient selection for BCG immunotherapy, this important finding of the present study might promise hope for the management of NMIBC cases by using a very cheap and easy technique. A better antitumour activity of BCG in NMIBC cases with decreased DNA MMR gene expression may be related to the cancer neoantigens caused by tumour gene mutations accumulating as a result of DNA repair defects [44].

## 5. Conclusion

Our study is the first to investigate the role of DNA MMR expression, microsatellite instability, and PD-L1 expression, all together, in the BCG failure of NMIBC cases. Our findings suggest that the PD-1/PD-L1 mediated immune escape mechanism may have a role as an underlying mechanism in the progression of urothelial carcinomas, whereas it does not seem to have any role in the BCG failure. Our study revealed that the evaluation of DNA MMR protein expression may be useful in predicting NMIBC cases that will respond well to BCG therapy and a better antitumour activity of BCG in NMIBC cases with decreased DNA MMR gene expression may be related to the cancer neoantigens caused by tumour gene mutations accumulating as a result of defective DNA repair. Since there is no validated predictive biomarker to guide patient selection for BCG immunotherapy, this important finding of the present study might promise hope for the management of NMIBC cases by using a very cheap and easy technique. However, our study had some limitations such as relatively small number of cases and retrospective design. Further studies with larger case series may contribute to our findings.

## Figures and Tables

**Table 1:** Patient demographics.

Clinicopathological features	Total n (%)	BCG responsive group n (%)	BCG nonresponsive group n (%)	p-value
**Sex**				
Female	6 (10.1)	5 (16.7)	1 (3.4)	0.195
Male	53 (89.8)	25 (83.3)	28 (96.6)	
**Multifocality**				
Solitary	26 (44)	19 (63.4)	7 (24.1)	0.001
Multiple	32 (54.3)	10 (33.3)	22 (75.9)	
Missing Data	1 (1.7)	1 (3.3)	0 (0)	
**Tumour size**				
< 3cm	13 (22)	7 (23.3)	6 (20.7)	0.7529
≥ 3cm	45 (76.3)	22 (73.4)	23(79.3)	
Missing data	1 (1.7)	1(3.3)	0 (0)	
**Nuclear pleomorphism**				
Mild	27 (45.8)	15 (50)	12 (41.4)	0.506
Prominent	32 (54.2)	15 (50)	17 (58.6)	
**Presence of concomitant carcinoma in situ (CIS)**				
Absent	49 (83)	25 (83.3)	24 (82.8)	1.000
Present	10 (17)	5 (16.7)	5 (17.2)	
**Tumour stage (pT)**				
pTa	8 (13.6)	3 (10)	5 (17.2)	0.472
pT1	51 (86.4)	27 (90)	24 (82.8)	
**Depth of lamina propria invasion in pT1 patients**				
Superficial l. propria	33 (64.7)	21 (77.8)	12 (50)	0.038
Deep l. propria	18 (35.3)	6 (22.2)	12 (50)	
**The extent of invasion in pT1 patients**				
Focal (1 Focus)	28 (55)	15 (55.6)	13 (54.1)	0.774
Multifocal (>1 Focus)	23 (45)	12 (44.4)	11 (45.8)	
**Long diameter of largest invasion focus in pT1 patients**				
<2 mm	32 (62.7)	18 (66.7)	14 (58.3)	0.585
≥2 mm	19 (37.3)	9 (33.3)	10 (41.7)	

**Table 2:** The distribution of ICP groups and PD-L1 expression of IC in pretreatment TUR-B specimens of both BCG nonresponsive and BCG responsive cases (n = 59).

Evaluation	n (%)	BCG responsive group n (%)	BCG nonresponsive group n (%)	p-value
ICP group 1 (≤5% ICP)	34 (57.6)	17 (56.6)	15 (51.7)	0.703
ICP group 2 (6%–10% ICP)	13 (22)	8 (26.6)	6 (20.6)	
ICP group 3 (>10% ICP)	12 (20.4)	5 (16.8)	8 (27.7)	
Low PD-L1 in IC	25 (42.4)	12 (40)	13 (44.8)	0.708
High PD-L1 in IC (≥25%)	34 (57.6)	18 (60)	16 (55.2)	

**Table 3:** Comparison between pretreatment and posttreatment TUR-B specimens of BCG nonresponders in terms of PD-L1 expression status.

PD-L1 expression in pretreatment and posttreatment TUR-B specimens of BCG nonresponders	n (%)
Cases with high PD-L1 expression in the pretreatment TUR-B specimen and negative/low PD-L1 expression in the posttreatment TUR-B specimen	6 (22.2)
Cases with negative/low PD-L1 expression in the pretreatment TUR-B specimen and high PD-L1 expression in the posttreatment TUR-B specimen	3 (11.1)
Cases with negative/low PD-L1 expression in both of pretreatment and posttreatment TUR-B specimens	8 (29.7)
Cases with high PD-L1 expression in both of pretreatment and posttreatment TUR-B specimens	10 (37)

**Table 4:** The relationship between reduced DNA MMR protein expression and response to BCG therapy (p = 0.022).

DNA MMR protein expression	BCG responsive group (n = 30) n (%)	BCG nonresponsive group (n = 29) n (%)	Total (n = 59) n (%)
**Reduced expression**	**17 (70.8)**	**7 (29.2)**	**24 (100)**
Isolated MSH6	8 (72.7)	3 (27.3)	11 (100)
Isolated PMS2	5 (83.3)	1 (16.7)	6 (100)
MSH2 and MSH6	0 (0)	1 (100)	1 (100)
PMS2 and MSH6	3 (60)	2 (40)	5 (100)
PMS2, MSH2, and MSH6	1 (100)	0 (0)	1 (100)
**Preserved expression**	13 (37.2)	22 (62.8)	35 (100)
